# DTD, an anti-inflammatory ditriazine, inhibits angiogenesis *in vitro* and *in vivo*

**DOI:** 10.1111/j.1582-4934.2008.00147.x

**Published:** 2008-08-11

**Authors:** Beatriz Martínez-Poveda, Ramón Muñoz-Chápuli, Ricardo Riguera, Antonio Fernández, Miguel Ángel Medina, Ana R Quesada

**Affiliations:** a Departamento de Biología Molecular y Bioquímica, Facultad de Ciencias, Universidad de MálagaMálaga, Spain; b Departamento de Biología Animal, Facultad de Ciencias, Universidad de MálagaMálaga, Spain; c Departamento de Química Orgánica, Universidad de SantiagoSantiago de Compostela, Spain; d Instituto Biomar S.A. Políg. Ind., edificio CEIOnzonilla, León, Spain

**Keywords:** angiogenesis inhibitor, DTD, ditriazines, endothelial cells

## Abstract

The ditriazine derivative DTD (4,10-dichloropyrido[5,6:4,5]thieno[3,2-d':3,2-d]-1,2,3-ditriazine) has been previously reported to reduce the degree of granulomatous inflammation and vascular density in a murine air pouch granuloma model. The aim of this study was to test whether DTD affects angiogenesis. Our results show that DTD inhibits *in vivo* angiogenesis in the chorioallantoic membrane (CAM) assay at doses equal or lower than 0.3 nmol/egg. Different *in vitro* assays were used to study the potential effects of this compound on key steps of angiogenesis, namely, a colorimetric assay of cell proliferation/viability, a morphogenesis on Matrigel assay, zymographic assays for gelatinases and nuclear morphology and cell cycle analysis for apoptosis induction. Our data indicate that DTD inhibits proliferation but does not induce apoptosis in endothelial cells *in vitro*. DTD suppresses the endothelial capillary-like chord formation at concentrations lower than those required to inhibit proliferation. DTD treatment inhibits the matrix metalloproteinase-2 production in endothelial and fibrosarcoma cells, but does not affect the cyclooxygenase-2 expression in endothelial cells, as assessed by western blot analysis. Taken together, results here presented indicate that DTD exhibits an anti-angiogenic activity that is independent of inflammatory processes and make it a promising drug for further evaluation in the treatment of angiogenesis-related pathologies.

## Introduction

Angiogenesis, the generation of new capillaries through a process of pre-existing microvessel sprouting, is under stringent control and normally occurs only during embryonic and post-embryonic development, reproductive cycle and wound healing. However, in a number of pathological conditions including tumour progression, metastasis, diabetic retinopathy, age-related macular degeneration, haemangioma, arthritis and psoriasis among others, the disease appears to be associated with persistent up-regulated angiogenesis. For this reason the development of specific anti-angiogenic agents arises as an attractive therapeutic approach for the treatment of cancer and other angiogenesis-dependent diseases [1, 2]. Angiogenesis has been described as one of the hallmarks of cancer, playing an essential role in tumour growth, invasion and metastasis [3]. This has driven the field of angiogen-esis research, considered to be able to change the face of medicine in the next decades [4]. After an initial pessimism derived from the modest or even negative results obtained with the first generation of inhibitors of angiogenesis entered in clinical trials, a second generation of anti-angiogenic therapies is showing positive results in phases II and III trials, including the Food and Drug Administration approval for several anti-angiogenic-based therapies in oncology and ophthalmology [5–7].

**Fig. 1:**
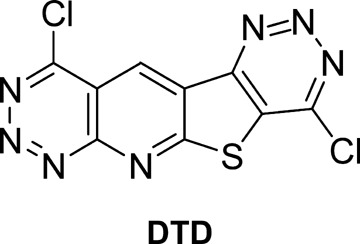
Chemical structure of DTD.

The formation of new blood vessels is a complex multi-step process, related to changes in endothelial cell biosignalling [8]. Once the ‘angiogenic switch’ is connected, endothelial cells resting in the parent vessels are activated and stimulated to synthesize and release degradative enzymes allowing endothelial cells to migrate, proliferate and finally differentiate to give rise to capillary tubules. Any of these steps may be a potential target for pharmacological intervention [9]. 4,10-dichloropyrido[5,6:4,5]thieno[3,2-d':3,2-d]-1,2,3-ditriazine (DTD) (Fig. 1), is a ditriazine derivative that modulates acute inflammation in murine models by inhibition of leukocyte functions and expression of nitric oxide synthase (NOS) and cyclooxygenase-2 (COX-2) [10]. Oral administration of DTD reduced the degree of granulomatous inflammation and vascular density in a murine air pouch granuloma model. The inhibition of the endogenous production of angiogenic cytokines and COX-2 expression in the granuloma, was then suggested to participate in the inhibition of vascularization by DTD in this model of inflammation [11].

Results presented here show for the first time that the antiangiogenic effect of DTD is not only due to a putative modulation of the production of angiogenic cytokines in inflammation, but it is exerted directly on endothelial cells. DTD inhibits angiogenesis *in vivo* in a widely employed angiogenesis assay (chorioallantoic membrane assay). Furthermore, *in vitro* assays show that DTD interferes several functions of the activated endothelial cells, namely proliferation, proteases production and differentiation. Taken together, our data suggest the possibility of utilising this compound for the treatment of angiogenesis-related diseases.

## Materials and methods

### Materials

Cell culture media were purchased from Gibco (Grand Island, NY, USA) and Cambrex (Walkersville, MD, USA). Foetal bovine serum (FBS) was a product of Harlan-Seralab (Belton, U.K.). Matrigel was purchased from Becton Dickinson (Bedford, MA, USA). Supplements and other chemicals not listed in this section were obtained from Sigma Chemicals Co. (St. Louis, Mo., USA). Plastics for cell culture were supplied by NUNC (Roskilde, Denmark). DTD was prepared by diazotation of 3,6-diamino-2,5-dicyanothieno[2,3-b]pyridines according to modified procedures from the literature [12], dissolved in dimethylsulfoxide (DMSO) at a concentration of 2 mg/ml and stored at −20°C until use. COX-2 monoclonal antibody was purchased from Santa Cruz Biotechnology (Santa Cruz, California, USA), the peroxidase-conjugated antimouse IgG was purchased from Amersham Biosciences (Buckinghamshire, UK), and the β-actin, monoclonal antibody was from Sigma. Fertilised chick eggs were obtained from Granja Santa Isabel (Córdoba, Spain).

### Cell cultures

HT1080 human fibrosarcoma cells were maintained in Dulbecco's modified Eagle's medium (DMEM) containing glucose (4.5 g/l), glutamine (2mM), penicillin (50 IU/ml), streptomycin (50 μg/ml), and amphoterycin (1.25 μg/ml) supplemented with 10% FBS (DMEM/10%FBS). HL60 human leukaemia cells were grown in RPMI 1640 medium supplemented with 10% (v/v) FBS. Bovine aortic endothelial (BAE) were grown in low glucose (1 g/l) DMEM/10% FBS. Human umbilical vein endothelial (HUVE) cells were isolated from human umbilical chords by collagenase digestion [13] and maintained in Medium 199 containing HEPES (10 mM), L-glutamine (2 mM), heparine (10 mg/mL), penicillin (50 IU/ml), streptomycin (50 μg/ml) and amphotericin (1.25 μg/ml), supplemented with 3 mg/l endothelial cell growth supplement (ECGS, Sigma) and 20 % FBS in 5% CO_2_ and 37°C.

### Endothelial cell differentiation assay: chord formation on Matrigel

Wells of a 96-well plate were coated with 50 μL of Matrigel (10.5 mg/ml) at 4°C and allowed to polymerise at 37°C for a minimum of 30 min [14]. 5 × 10^4^ BAE cells were added in 200 μl of DMEM. For HUVEC, 2.5 × 10^4^ cells were added in 200 μl Medium 199 supplemented with 5% FBS. Finally, different amounts of DTD were added and incubated at 37°C in a humidified chamber with 5% CO_2_. After incubation for 7 hrs, cultures were observed (200 **x** magnification) and photographed with a NIKON inverted microscope DIAPHOT-TMD (NIKON Corp., Tokyo, Japan). Each concentration was tested in duplicate, and two different observers evaluated the results of chord formation inhibition. Those assays where no tubular structure could be observed were considered as positive in the inhibition of morphogenesis of endothelial cell on Matrigel.

### Cell growth assay

The 3-(4,5-dimethylthiazol-2-yl)-2,5-diphenyltetrazolium bromide (MTT; Sigma Chemical Co., St. Louis, MO) dye reduction assay in 96-well microplates was used, as previously described [15]. The assay is dependent on the reduction of MTT by mitochondrial dehydrogenases of viable cell to a blue formazan product, which can be measured spectrophotometrically. 3 × 10^3^ BAE, 4 × 10^3^ HUVE, 2 × 10^3^ HL60 and HT-1080 in a total volume of 100 μl of their respective growth media were incubated with serial dilutions of DTD. After 3 days of incubation (37°C, 5% CO_2_ in a humid atmosphere) 10 μl of MTT (5 mg/ml in phosphate-buffered saline [PBS]) were added to each well and the plate was incubated for a further 4 hrs (37°C). The resulting formazan was dissolved in 150 μL of 0.04 N HCl-2 propanol and read at 550 nm. All determinations were carried out in triplicate. IC50 value was calculated as the concentration of DTD yielding a 50% of cell survival.

### Hoechst staining

BAE cells were plated on coverslips and grown to 75% confluence. After treatment with DTD at 3 and 15 μM during 14 hr, cells were fixed with formalin solution (Sigma), washed with PBS and stained with 1 μg/ml Hoechst in PBS. Coverslips were mounted on slides using Dakocytomation Fluorescent Mounting Medium (Dako) and observed under a fluorescence microscope (Leica, TCS-NT).

### Cell cycle analysis

After treatment with DTD at 3 and 15 μM during 14 hrs, attached and detached BAEC were harvested and centrifuged. Pellets were washed with PBS and resuspended in 250 μl of ice-cold PBS. For fixation, 70% ice-cold ethanol was added while continuous gentle vortexing and samples were maintained on ice for 1 hr. Finally, cells were centrifuged and washed twice with PBS, re-suspended in 500 μl propidium iodide staining solution (40 |Ag/ml propidium iodide and 0.1 mg/mL RNase-A in PBS) and incubated during 1 hr at 37°C protected from light. Percentage of subG1 G1, S and G2/M cells was determined using a MoFlo DakoCytomation cytometer.

### Chorioallantoic membrane (CAM) assay

CAM assay was performed as described [16]. Fertilised chick eggs were incubated horizontally at 38°C in a humidified incubator, windowed by day 3 of incubation and processed by day 8. DTD was added to a 0.7% solution of methylcellulose in water, and 10 μl drops of this solution were allowed to dry on a Teflon-coated surface in a laminar flow hood. After implanting the methylcellulose discs on the CAM, the eggs were sealed with adhesive tape and returned to the incubator for 48 hrs. Negative controls were made with DMSO mixed with the methylcellulose. After re-incubation, the CAM was examined under a stereomicroscope by two different observers. The assay was scored as positive when both of them reported a significant reduction of vessels in the treated area.

### Conditioned media and gelatinograms

To prepare conditioned media, HUVE and HT-1080 cells were grown in 6-well plates. When the cells were at 75% confluency, medium was aspirated, cells were washed twice with PBS and each well received 1.5 ml of DMEM/0.1% BSA containing 200 KIU of Aprotinin/ml. Additionally, some wells received DTD 3 and 15 μM. After 24 hrs of incubation, conditioned media were collected and centrifuged at 1000 × gand 4°C for 20 min and used for zymography. Cell number was determined by using a Coulter counter.

The gelatinolytic activities of matrix metalloproteinase-2 (MMP-2) and matrix metalloproteinase-9 (MMP-9) delivered to the conditioned media were detected as previously described [17]. Aliquots of conditioned media normalized for equal cell numbers were subjected to non-reducing SDS-PAGE with gelatin (1mg/ml) added to the 10% resolving gel. After electrophoresis, gels were washed twice with 2% Triton X-100 in 50 mM Tris/HCl, pH 7.4, and twice with 50 mM Tris/HCl, pH 7.4. Each wash was for 10 min and with continuous shaking. After incubation (overnight at 37°C) in a substrate buffer (50 mM Tris/HCl, pH 7.4, 1% Triton X-100, 5 mM CaCl_2_, and 0.02% Na_3_N), gels were stained with Coomassie blue R-250 and the bands of gelatinase activity were detected as non-stained bands in a dark, stained background.

### Western-blot analysis

Sub-confluent HUVE cells were stimulated with 50 ng/ml PMA (phorbol 12-myristate 13-acetate) during 4 hrs 30 min. At the same time, cells were treated with DTD 12 μM. Cell culture dishes were briefly washed twice with ice-cold PBS before adding lysis buffer (50 mM Tris, pH7.4, 150 mM NaCl, 1%Triton X-100, 0.25% sodium deoxycholate, 1 mM ethylenediaminetetraacetic acid (EDTA), 1 mM sodium orthovanadate, 30 mM β-glyrerophosphate, 30 mM sodium fluoride, 5 mM disodium pyrophosphate, 5 μl/ml protease inhibitor cocktail). After scraping lysates were kept on ice for 15 min and centrifuged at 13,000 rpm during 15 min at 4°C. A minimum of 25 μg of total protein of each sample were subjected to electrophoresis with 10% SDS-PAGE and transferred to nitrocellulose membranes. Membranes were blocked in Tris 20 mM NaCl 137 mM, Tween-20 0.1%, 10% skimmed milk, pH 7.6 (blocking buffer) and incubated overnight with the IgG monoclonal antibody to COX-2 (1/500). After washing, membranes were incubated with the horseradish peroxidase-conjugated sheep antimouse IgG (1/5000) in blocking buffer and the immunoreactive bands were visualized using an enhanced chemiluminiscence system (SuperSignal Wets Pico chemilu-miniscent substrate, Pierce, Rockford, IL, USA). For detection of β-actin IgG monoclonal antibody was used.

## Results

### DTD inhibits *in vivo* angiogenesis in the chick chorioallantoic membrane assay

**Fig. 2:**
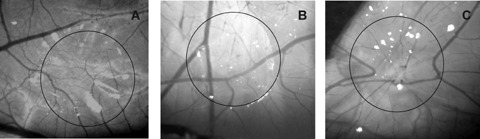
Chorioallantoic membrane assay of DTD. 0.6 nmol (Panel B) and 3 nmol (Panel C) of DTD were used in this assay. In both cases pre-existing vessels were disorganized and peripheral vessels grew centrifugally, avoiding the treated area. Panel A shows a control methylcellulose disc containing the substance vehicle alone. Circles show the locations of the methylcellulose discs.

The CAM assay is frequently used to determine the ability of test compounds to inhibit *in vivo* angiogenesis. In controls, blood vessels form a dense and spatially oriented branching network composed by vascular structures of progressively smaller diameter as they branch (Fig. 2A). Table 1 summarises the evaluation of the *in vivo* inhibition of angiogenesis in the CAM assay by DTD, showing that the anti-angiogenic activity of this compound is maintained as low as 0.3 nmol per CAM, where 80% of the eggs scored positive. DTD anti-angiogenic effect was observed as an inhibition of the ingrowth of new vessels in the area covered by the methylcellulose discs. The peripheral vessels (relative to the position of the disc) grew centrifugally, avoiding the treated area, where a decrease in the vascular density could be observed (Fig. 2B and C). Signs of inflammation, such as a whitening of the CAM, were not observed.

**Table 1:** Inhibition of *in vivo* angiogenesis by DTD

Dose (nmol/CAM)	Positive/Total	% positive
0	0/20	0
0.15	0/3.	0
0.3	4/5	80
0.6	3/4	75
1.5	3/4	75
3	4/4	100
6	3/3	100

CAM assay was carried out with different doses of DTD, as described in Material and methods. Data are given as percentage of eggs with inhibited angiogenesis in their CAMs per total number of treated egg CAMs

### DTD inhibits the growth of endothelial and tumour cells

**Fig. 3:**
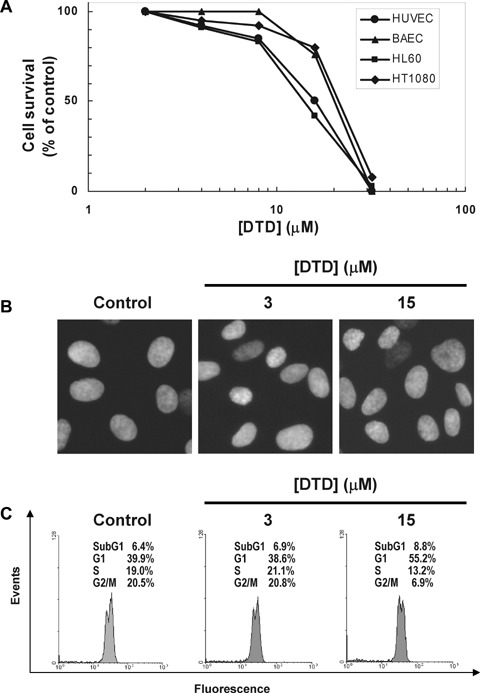
(**A**) Dose-dependent effect of DTD on the *in vitro* growth of human umbilical vein endothelial (HUVE) (•), BAE (▴), HL60 (▪) and HT1080 (♦) cells. Cell proliferation is represented as a percentage of control-cells growth in cultures containing no drug. Each point represents the mean of triplicates; SD values were always lower than 10% of the mean values and are omitted for clarity. (**B**) Effect of DTD on endothelial nuclear morphology. BAEC were grown on covers, treated with the indicated concentrations of DTD for 14 hrs, fixed with formalin and stained with Hoechst 1 μg/ml. Covers were mounted on slides and nuclei were observed under fluorescence microscope. (**C**) Effect of DTD on endothelial cell cycle distribution. BAEC were exposed for 14 hrs to DTD at the indicated concentrations, stained with propidium iodide and percentage of subG1, G1, S and G2/M cells were determined using a MoFlo DakoCytomation cytometer. Representative of one of two experiments done with superimposable results.

Angiogenesis involves local proliferation of endothelial cells. We investigated the ability of DTD to inhibit the growth of endothelial cells. Survival curves obtained with the MTT assay showed that DTD inhibited, in a concentration-dependent manner, the growth of cultured endothelial cells (Fig. 3A). IC50 value of this anti-proliferative effect was 21.5±1.8 μM and 14.5±2.5 μM (means of three different experiments ± S.D.), for BAE and HUVE cells, respectively. Data obtained with HT1080 and HL60 tumour cells lines show that the effects of DTD on cell growth are not specific for endothelial cells (Fig. 3A).

### DTD does not induce apoptosis in endothelial cells

As a first approach to determine whether DTD could induce apoptosis in endothelial cells, nuclear morphology and flow cytometric analysis of the cell sub-population distribution were investigated in BAE cells after 14 hrs treatment with 3 or 15 μM of DTD. Our data show that neither significant changes on the nuclear morphology (Fig. 3B) nor significant increases in sub-G1 population (Fig. 3C) were observed in endothelial cells treated with 3 or 15 μM DTD, suggesting that DTD does not induce apoptosis in endothelial cells. No significant effect on cell cycle distribution was observed when BAE cells were treated with 3 μM DTD. However, flow cytometric analysis showed that DTD treatment (15 μM) for 14 hrs resulted in an appreciable decrease of BAEC in the S and G2/M phases of cell cycle (13.2 and 6.9% versus 19.0 and 20.5% of control, respectively). This decrease in the populations of S and G2/M cells was accompanied by a concomitant increase in cell number in the G1 phase (55.2% versus 39.9% of control) (Fig. 3C). This pattern is consistent with blocks to progression at the S and G2/M phases of the cell cycle and may underlie the growth inhibitory activity observed for this compound.

## DTD inhibits the capillary chord formation by endothelial cells

**Fig. 4:**
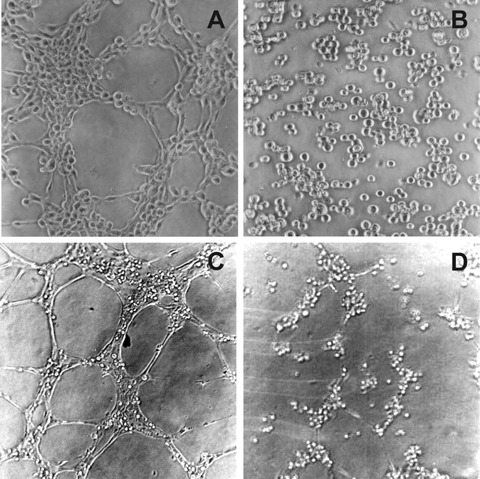
Effect of DTD on endothelial cell tubulogenesis *in vitro*. BAE and HUVE cells seeded on Matrigel formed chords (**A** and **C**, respectively). Addition of DTD (Panel B, 1.5 μM DTD BAEC; **D**, 3 μM DTD HUVEC) inhibited tubulogenesis. Cells were photographed under an inverted microscope (×200) 7 hrs after seeding.

The final event during angiogenesis is the organisation of endothelial cells in a three-dimensional network of tubes. *in vitro*, endothelial cells plated on Matrigel align themselves forming tubelike structures, already evident a few hours after plating (Fig. 4A and C) Figures 4B and 4D show that DTD was able to completely inhibit the BAE and HUVE cell alignment and chord formation. The minimal concentration of compound yielding a complete inhibition of endothelial morphogenesis on Matrigel was 1.5 μM for BAE and 3.0 μM for HUVE cells. The concentrations required to inhibit the differentiation of BAE and HUVE cells, did not affect their viability after 7 hrs (results not shown).

### Effect of DTD on endothelial and tumour cells gelatinases secretion

**Fig. 5:**
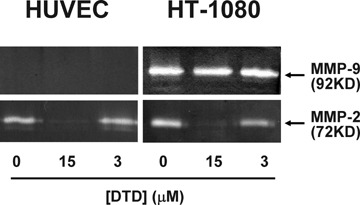
Effect of DTD on endothelial and tumour cells gelatinases secretion. Conditioned media of HUVE and HT1080 treated with 3 and 15 μM DTD and non-treated cells (control) were normalized for equal cellular density and used for gelatin zymography as indicated in the Methods section.

Angiogenesis involves the acquisition by endothelial cells of the capability to degrade the basement membrane and, in general, to remodel the extracellular matrix. As shown in Fig. 5, in our hands HUVE cells express the 72 kD pro-form of MMP-2. However, no MMP-9 secretion by HUVEC could be detected. Gelatin zymography of conditioned media of HUVE cells untreated and treated for 24 hrs with 3 or 15 μM of DTD (Fig. 5), shows that 15 μM of this compound produced complete inhibition of MMP-2 secretion by endothelial cells. The effect on MMP-2 production does not seem to be endothelial-specific, since a decrease in MMP-2 secretion by HT1080 tumour cells was also observed after treatment with 15 μM DTD. No effect on MMP-9 levels was observed after HT1080 treatment with 15 μM DTD (Fig. 5).

### Effect of DTD on endothelial COX-2 expression

**Fig. 6:**
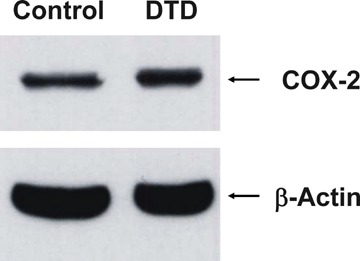
DTD (15 μM) does not affect the expression of COX-2 by HUVEC.

Inhibition of either endothelial COX-2 expression or of its enzymatic activity has recently been considered a new strategy to inhibit angiogenesis. Taking into account the previously described down-regulation of COX-2 expression levels by DTD in inflammatory cells [10, 11], the effect of DTD on endothelial COX-2 expression was investigated. As shown in Fig. 6, 15 μM DTD did not affect the expression level of COX-2 in HUVE cells.

## Discussion

The new ditriazine derivative DTD exerts anti-inflammatory effects related to the inhibition of neutrophil functions and of NO and prostaglandin E2 (PGE_2_) production, which could be due to a decreased expression of inducible NO synthase and COX-2 in activated macrophages [10]. DTD inhibits the vascularisation in an inflammatory model, what has been suggested to be related to inhibition of cytokine and PGE2 production by interfering with NF-κB activation [11]. The objective of our work has been to determine if DTD could inhibit angiogenesis *in vivo* in a model where inflammation was not directly involved, and to elucidate if DTD might directly interfere any of the following key steps mediated by endothelial cells: degradation of the basement membrane, proliferation of endothelial cells, and the formation of capillary-like chords.

The evaluation of the potential anti-angiogenic activity of test compounds in the *in vivo* CAM assay is one of the most widely used *in vivo* angiogenesis assays [18]. Our results show that DTD inhibits the neovascularisation of the chick chorioallantoic membrane at concentrations that are several orders of magnitude lower than those required for other inhibitors of angiogenesis [19–21], demonstrating that this compound exhibits a potent anti-angiogenic activity, independently of a putative modulation of the production of angiogenic cytokines in an inflammatory environment.

Capillary endothelial cells proliferate in response to an angiogenic stimulus during neovascularisation. Our data show that DTD inhibits the growth of endothelial cells by slowing down proliferation. DTD caused accumulation of endothelial cells in the G1 phase of the cell cycle, with concomitant depletion of S and G2/M phases of the cell cycle, while inducing little or no apoptosis. IC50 values obtained with DTD in BAE and HUVE cells are higher than those reported for compounds to be considered to exert their anti-angiogenic activity by inhibition of endothelial cell proliferation [22, 23]. DTD is not a specific inhibitor or endothelial cell growth. This lack of specificity is also exhibited by other anti-angiogenic compounds that are considered to act mainly through inhibition of endothelial cell proliferation [23]. Although in our hands the anti-proliferative activity has not been the most prominent effect of DTD on endothelial cells, its contribution to the anti-angiogenic potential role of DTD can not be ruled out.

Our data indicate that DTD inhibits capillary-like chord formation by endothelial cells at concentrations that are lower than or in the same range as those required for other previously described inhibitors of angiogenesis [19–21, 24, 25]. The concentrations required for a complete abrogation of tubulogenesis were lower than those required to inhibit cell proliferation, and do not affect human neutrophils or murine macrophages viability [10, 11]. Therefore, although a role of the anti-proliferative activity of DTD could not be discarded, our results suggest that DTD anti-angiogenic activity could be dependent on prevention of capillary-like chord formation rather than endothelial cell proliferation. Recently Miller et al. [26] have suggested criteria by which a chemotherapeutic agent might reasonably be considered to have meaningful anti-angiogenic activity. Taking into account that DTD interferes with endothelial cell differentiation at concentrations that do not cause cell death, this compound could be considered an anti-angiogenic compound, assessment that is reinforced by the angiogenesis inhibitory activity of DTD *in vivo*. It should be pointed out that the previously reported effects of DTD on the functions of inflammatory cells were also exerted at concentrations that did no affect their viability [3, 4].

A positive proteolytic balance is required for capillary sprouting and lumen formation during angiogenesis. Matrix metallo-proteinases, particularly the gelatinases MMP-2 and MMP-9, play a central role during angiogenesis [27]. Endothelial cells constitutively secrete MMP-2, which is required for the tumour to trigger the angiogenic response [28]. MMP-2 is secreted as an 72 kD inactive pro-form; when it is converted to a 62 kD active form it can degrade collagen types IV and V, laminin and elastin. In intact cells, MMP-2 is activated at the cell surface by a process involving interaction of the C-terminal component of MMP-2 with a plasma membrane activation mechanism [27]. Our data show that incubation with DTD inhibits MMP-2 pro-form secretion by HUVE cells. Similar decreases of pro-MMP-2 levels in the conditioned media of endothelial cells have been described for halofuginone [29] and aeroplysinin-1 [24], and they have been suggested to lead to the inhibition of the endothelial cell tube formation *in vitro*. This is in agreement with previously reported data showing that when endothelial cells are cultured on Matrigel, the formation of tubular networks is increased by the addition of recombinant MMP-2 and decreased when a neutralizing antibody is added [30]. The inhibition of MMP-2 production by DTD does not seem to be endothelial-spe-cific, since a similar effect was observed in HT-1080 fibrosarcoma cells. However MMP-9 secretion by HT1080 cells is not affected by DTD incubation. MMP-9 is not constitutively expressed by endothelial cells, but it may be induced in response to several factors, including the tumour promoter chemical phorbol myristate acetate (PMA), cytokines or stress [27, 31, 32]. Our data show that incubation with DTD does not induce the 92 KD pro-MMP-9 secretion by HUVE cells.

The zinc-finger transcription factor Ets-1 seems to play a key role in activation of the proteolytic system by transactivation of the promoters of many proteases, including the MMPs. Expression of Ets-1 is mediated by the mitogen activated protein kinase (MAPK, ERK1 and ERK2) pathway. The effect of DTD on MMP-2 expression could suggest a possible intervention of this pathway, what could also explain DTD inhibition of the endothelial cell morphogenesis and proliferation, also mediated by the MAPK/ERK pathway. On the other hand, a role of integrin-mediated pathways in the mechanism of action of DTD can not been discarded, since they are involved in the transduction of the signals leading to the proliferation, differentiation and extracellular matrix degradation by endothelial cells [8].

Cyclooxygenase-2 (COX-2), a key enzyme in the synthesis of prostaglandins and thromboxans, is highly up-regulated in tumour cells, stromal cells and angiogenic endothelial cells during tumour progression. The contributions of COX-2 in tumour angiogenesis include: (a) the increased expression of the proan-giogenic growth factors; (b) the production of the eicosanoid products thromboxaneA2, PGE_2_ and PGI_2_ that can directly stimulate endothelial cell migration and growth factor-induced angiogenesis; and potentially, (c) the inhibition of endothelial cell apoptosis by stimulation of Bcl-2 or Akt activation [33]. Therefore, the targeting of endothelial COX-2, either by inhibiting its enzymatic activity or by blocking its transcription, might be useful in combating angiogenesis-dependent diseases [34]. Previous results showing that DTD inhibited the COX-2 expression by lipopolysaccharide-stimulated murine peritoneal macrophages *in vitro*[10] and that a decreased COX-2 expression was observed in murine air pouch granuloma after oral administration of DTD [11], suggested the possibility that the anti-angiogenic activity of DTD could be also due to a direct modulation of the endothelial COX-2 expression, what could induce endothelial apoptosis cells by inhibition of the Akt signalling axis [35]. Our results show that incubation with DTD neither affect the expression of COX-2 in HUVE cells, nor induces detectable apoptosis in endothelial cells, suggesting that different signalling pathways are modulated by DTD in endothelial and inflammatory cells.

In conclusion, we have shown for the first time that DTD is able to inhibit endothelial cell proliferation, differentiation and MMP-2 secretion *in vitro*, and it exhibits a potent inhibition of *in vivo* angiogenesis in the chick chorioallantoic mem-brane. Although additional studies will be needed to elucidate the molecular mechanisms underlying the anti-angiogenic activity of DTD, data presented here suggest its potential in therapeutic applications for the treatment of angiogenesis related diseases.
